# Sucrose alleviates capsaicin-induced tongue burning: An *in vivo* study

**DOI:** 10.4317/jced.58911

**Published:** 2022-07-01

**Authors:** Duangchewan Puengsurin, Rittinarong Meepong, Nattapon Rotpenpian, Aree Wanasuntronwong, Rudee Surarit

**Affiliations:** 1 Department of Oral Biology, Faculty of Dentistry, Mahidol University, Bangkok 10400, Thailand; 2 Faculty of Pharmaceutical Sciences, Burapha University, Chonburi 20131, Thailand; 3 Department of Oral Biology and Occlusion, Faculty of Dentistry, Prince of Songkla University, Songkhla, Thailand

## Abstract

**Background:**

Spicy foods are flavorful and stimulate salivation, which is beneficial for individuals with poor appetite. They are also ubiquitous in many regional cuisines, but the chemical compounds in such foods, especially capsaicin from chili peppers, can cause tissue inflammation and generate intolerable burning pain in the oral cavity.

**Material and Methods:**

To identify a potential method to reduce capsaicin-induced burning pain without influencing food flavor, we tested the effects of mouth rinsing with various concentrations of sucrose. Inclusion criteria were good general and oral health, while exclusion criteria were poor baseline smell or taste, capsaicin allergy, and current orofacial pain complaints. To define an appropriate capsaicin dose, participants placed filter paper strips impregnated with 0.003%–0.3% capsaicin on the tip of the tongue and rated burning sensation by visual analog scale (VAS) score.

**Results:**

A 0.1% capsaicin solution induced tongue burning in the midrange (VAS = 6.33 ± 0.52) and so was used for subsequent tests. We then examined the efficacy concentration of sucrose for reducing tongue burning by recording VAS scores at multiple time points following a 15-s oral rinse with various aqueous sucrose solutions (5%, 10%, and 20%), milk, or pure water (control) after 0.1% capsaicin application. Scores were compared at each time point by one-way ANOVA with post hoc Dunnett’s tests. A 15-s rinse with 20% sucrose significantly alleviated burning pain compared to water rinse at 45, 60, 120, and 180 s after capsaicin exposure.

**Conclusions:**

Thus, periodic rinsing with 20% aqueous sucrose may help promote spicy food consumption among individuals with poor appetite.

** Key words:**Capsaicin, sucrose, burning sensation.

## Introduction

Capsaicin (8-methyl-N-vanillyl-trans-6-nonenamide) is a hydrophobic compound found in the fruits of genus Capsicum (chili peppers). Chili peppers are used ubiquitously to add flavor and aroma to prepared foods, but capsaicin is an irritant to the oral cavity, producing both thermal (hot) and nociceptive (burning or stinging) sensations (1). Capsaicin activates transient receptor potential cation channel subfamily V member 1 (TRPV1) receptors on the unmyelinated C-fibers of primary afferent neurons in oral tissues. These neurons project to the brainstem trigeminal nucleus complex via maxillary and mandibular branches (V2 and V3) of the trigeminal nerve (1,2). Alvarez-Berdugo *et al*. found that TRPV1 receptor density was greater within the CN V region (tongue) epithelium than the CN X region (epiglottis) (3). Similarly, Tachibana and Chiba found that TRPV1 receptors are highly expressed in tongue epithelium, especially at the apex (4). Activation of TRPV1 receptors leads to depolarization, action potential generation, ensuing elevation of intracellular calcium (Ca2+), and release of neuropeptides such as substance *P* (SP) and calcitonin-gene related peptide (CGRP). Substance *P* induces vasodilation and activates histamine release. Similarly, CGRP also induces vasodilation, and collectively SP and CGRP modulate the transmission of heat and pain sensations (5,6). In 1980, Rozin and Schiller reported that humans with a preference for chili peppers were as sensitive to the burning sensation as people averse to chili peppers but nonetheless developed at taste for them (7). Conversely, Prescott and Stevenson (1995) reported that people who regularly consume foods with chili peppers reported less intense pain in response to a capsaicin solution than others stating a dislike for chili peppers (8).

In addition to pain and heat sensations, capsaicin also induces a robust parasympathetic response. Application of a high concentration of capsaicin to oral tissue was reported to induce salivary secretion, sweating, and other responses via a parasympathetic cholinergic reflex mechanism (8). Duner *et al*. reported that a capsaicin swab to the floor of the mouth and buccal mucosa induced salivation from submandibular and sublingual gland at 8 g/min, and salivation from the parotid gland at 4 g/min. The saliva stimulated by capsaicin also increased amylase secretion, which could aid in digestion (9).

Multiple studies have attempted to determine the lowest concentration of capsaicin detecTable by subjects (the capsaicin threshold). Lawless *et al*. reported a capsaicin threshold of 0.310 ppm in water and 11.75 ppm in oil (10). Similarly, Schneider *et al*. reported that the oral capsaicin threshold was lower in aqueous solution than in sunflower oil (0.080 vs. 0.826 ppm (11)). Further, higher capsaicin concentrations produced more intense pain. However, pain intensity is also dependent on the area stimulated, presumably reflecting TRPV receptor density. Simon *et al*. reported that application of 33 ppm (0.0033%) capsaicin onto the anterior dorsum of the tongue produced moderate pain (12), while Nasrawi and Pangborn reported that a 15-s whole-mouth rinsing with 3 ppm (0.0003%) capsaicin also produced moderate pain in subjects (VAS = 5) (13).

Several methods are used to alleviate the burning sensation from chili peppers, with cold beverage consumption the most common. However, the best method is still debated. Nasrawi and Pangborn found that rinsing the mouth with 10% sucrose solution at 20°C was as effective as whole milk at 5°C for reducing capsaicin-induced burning (13). Alternatively, Fitri *et al*. reported that the fat content of milk was more important than the sucrose content for reducing burning (14). Steven and Lawless found that citric acid and sucrose could reduce burning sensation quicker than NaCl, water, or quinine (15). Therefore, the objective of this study is to examine whether sucrose can reduce the tongue burning sensation in response to capsaicin.

## Material and Methods

-Study design, site, and participants

This experimental study was conducted from December 2016 to May 2017. The protocols were approved by the Ethics Committee of the Faculty of Dentistry and Faculty of Pharmacy, Mahidol University (approval No. COA MU-DT/PY-IRB 2016/064.2311). Written informed consent was obtained from all participants. The study was divided into two parts. First, the optimal dose of capsaicin for inducing reproducible and modifiable tongue burning pain (i.e., without floor and ceiling effects) was determined among a subset of five participants. Second, the effects of sucrose on capsaicin-induced tongue burning were investigated among 60 healthy volunteers. All participants were dental students from the Faculty of Dentistry, Mahidol University (ages 18-25 years). Exclusion criteria were systemic diseases, common cold or other disorders affecting gustation and olfaction, allergy to capsaicin, pregnancy, tooth hypersensitivity, current oral ulcer, complaints of orofacial pain or temporomandibular disorder, and intake of medications affecting the central nervous system.

-Capsaicin dose determination

A stock solution (0.3% w/v) of capsaicin (M2028, Sigma-Aldrich) was prepared by dissolving 0.3 mg in a 100 mL solution of deionized water and 10% TWEEN20 (Ajax Finechem, Australia). A pilot study was conducted to determine the optimal concentration of capsaicin for use in subsequent experiments. Six concentrations of capsaicin between 0.00001%–0.3% were tested on 5 individuals with a 30 min wash-out time between each test. A 1.2-cm diameter paper disk soaked in each capsaicin concentration was placed on the tongue tip and subjects rated the burning intensity using a visual analog scale (VAS), where 10 cm indicated the maximum burning sensation and 0 the absence of burning sensation.

-Effect of sucrose on capsaicin burning sensation

**Figure 1:**
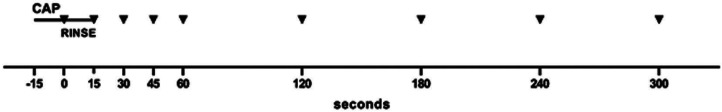
Experimental design and timeline. Capsaicin was applied on the tongue tip for 15 s, followed by a 15-s rinse. Subjects rated burning sensation on a VAS at 0, 15, 30, 45, 60, 120, 180, 240, and 300 s after removal of the capsaicin disk (inverted triangles).

Sucrose solutions were prepared at 5%, 10%, and 20% w/v in deionized water. Commercial UHT milk (Foremost) for testing was purchased in a retail store. All participants were asked not to eat spicy foods for at least 72 h before testing. Subjects were randomly allocated into five treatment groups: (i) pure deionized water (control), (ii) milk, (iii) 5% sucrose, (iv) 10% sucrose, and (v) 20% sucrose. A capsaicin-soaked paper disk was placed on the tip of tongue midline 1 cm from the apex for 15 s and subsequently, the burning sensation was recorded. Subjects rinsed their mouth once for 15 s with the assigned solution after capsaicin disk removal and burning sensation recorded immediately (15 s post-capsaicin) and again at 30, 45, 60, 120, 180, 240, and 300 s after capsaicin application (Fig. 1).

-Statistical analysis

Treatment group means were compared at each time point by one-way ANOVA with post hoc Dunnett’s tests for pair-wise comparisons. All tests were two-tailed and *P* < 0.05 was considered statistically significant.

## Results

-Optimal capsaicin test concentration

Average VAS ratings for different capsaicin concentrations are shown in Table 1. A VAS score between 5-7 was deemed optimal for subsequent tests because this level of burning sensation will not cause tissue injury but is reproducible and modifiable (with no floor or ceiling effects). Based on this criterion, a 0.1% capsaicin solution was used for all subsequent tests.

**Table 1:**
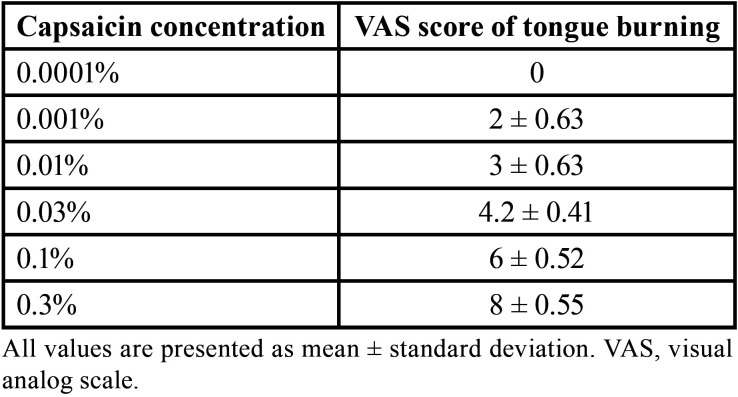
Average VAS scores for tongue burning pain induced by 0.00001%–0.3% capsaicin.

-Effects of rinsing solutions on capsaicin-induced burning sensation

**Figure 2:**
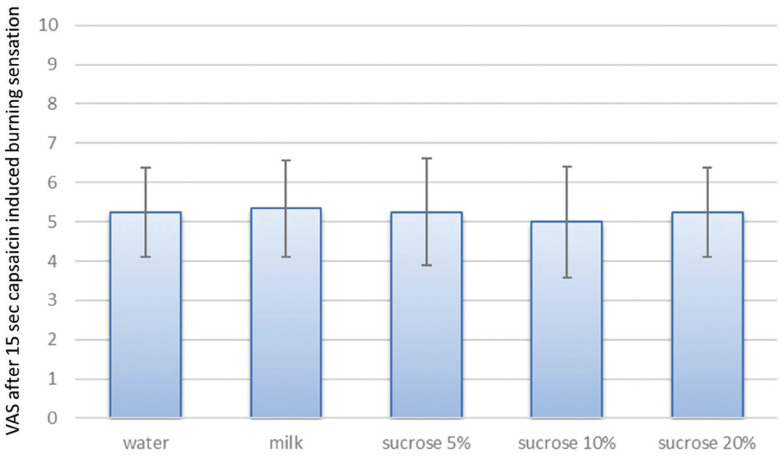
Capsaicin-induced burning pain in each group before rinsing with the test solution.

There were no significant differences in VAS score among groups immediately after removal of the capsaicin disk (Fig. 2). One minute after rinsing with the test solution, all groups reported lower VAS scores, but the difference was statistically significant only 45, 60, 120, and 180 s after rinsing with 20% sucrose ( Table 2, Table 3).

**Table 2:**
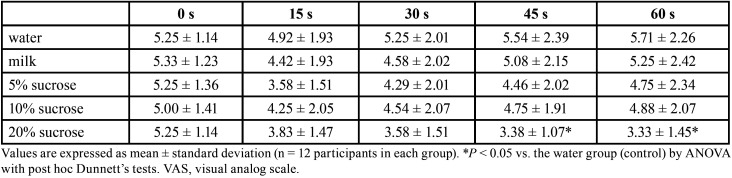
Short-term effects of each test solution on VAS score for capsaicin-induced pain.

**Table 3:**
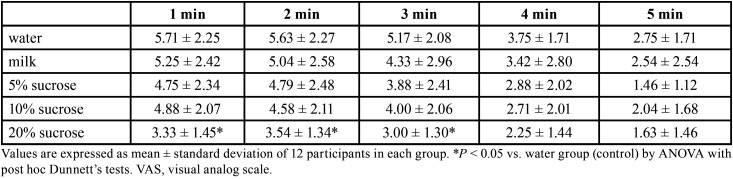
Longer-term effects of each test solution on VAS score for capsaicin-induced pain.

## Discussion

This study evaluated the effects of sucrose and milk on the oral burning sensation induced by capsaicin, the main sensory active compound in hot chili peppers. Results were in general agreement with previous studies (12,16) showing that rinsing with water cannot reduce this burning sensation, possibly due to the hydrophobicity of capsaicin. However, a previous study also found that a 5% ethanol rinse failed to reduce the burning sensation from capsaicin compared to water rinsing despite solubility in ethanol. Mouth rinsing with water or ethanol may temporarily lower the temperature of the oral cavity, and hence only transiently reduce the intensity of burning. Alternatively, Sizer and Harris reported that low temperature and sucrose concentration masked the burning sensation by raising the concentration threshold (17). This sucrose-induced reduction in pain sensation may result from a decrease in capsaicin binding to TRPV1 receptors. Table 2 shows that all sucrose concentrations suppressed burning caused by capsaicin, and that the effect was not strictly dose-dependent (10% was not uniformly more effective than 5%), and so was likely not due to saturation of capsaicin receptors. An alternative explanation is that the sensation of sweetness can suppress pain pathway transmission by reducing SP secretion in response to capsaicin (13). A recent study found that capsaicin receptors were co-localized with sweet- and bitter-sensing cells in the circumvallate papillae (18). In addition, application of sweet solution increased oral mucosal pain tolerance threshold in children (19). It is possible that activation of sweet receptors masks or interferes with the burning sensation induced by capsaicin receptor activation.

All groups except that rinsing with 20% sucrose demonstrated a progressive rise in VAS score 15 s to 60 s following capsaicin exposure (Table 2), possibly reflecting sensitization of peripheral nerves caused by SP release from nociceptive endings, leading to a buildup of inflammatory mediators that in turn depolarize nociceptive nerve endings (20). However, this sensitization did not occur following a rinse with 20% sucrose, likely because the more intense sweet sensation partially suppressed SP release (13). In the later period, pain sensation progressively decreased in all groups due to salivary clearance of capsaicin. Nonetheless, 20% sucrose solution still significantly reduced VAS score compared to water at 3 minutes. Thus, a sufficiently high sucrose concentration can reduce capsaicin-induced oral pain more effectively than lower concentrations.

These results indicate that 20% sucrose can reduce the oral burning pain caused by capsaicin. Further, sucrose can enhance taste. A previous study proposed that sweetness modulates the motivational-affective dimension of pain (21), possibly by reducing pain transmission to higher brain centers via C-fibers (22) and thereby increasing pain tolerance (22,23). However, the use of sucrose to reduce capsaicin-induced burning pain may be limited in some cases, such as for individuals with diabetes or abnormally low salivary flow rate as sucrose can exacerbate these disorders. Although many people prefer drinking milk to reduce capsaicin-induced burning, our study indicates that a high-concentration sucrose solution is more effective. Sucrose is one of the main sweeteners in commercially available beverages. Therefore, we recommend 20% sucrose solution for reducing tongue burning pain from capsaicin, especially for people intolerant of dairy products.
